# Big Cat Coalitions: A Comparative Analysis of Regional Brain Volumes in Felidae

**DOI:** 10.3389/fnana.2016.00099

**Published:** 2016-10-20

**Authors:** Sharleen T. Sakai, Bradley M. Arsznov, Ani E. Hristova, Elise J. Yoon, Barbara L. Lundrigan

**Affiliations:** ^1^Department of Psychology, Michigan State University, East LansingMI, USA; ^2^Neuroscience Program, Michigan State University, East LansingMI, USA; ^3^Department of Psychology, Minnesota State University, Mankato, MankatoMN, USA; ^4^Department of Integrative Biology and Michigan State University Museum, Michigan State University, East LansingMI, USA

**Keywords:** endocast, frontal cortex, computed tomography, lion, cheetah, leopard

## Abstract

Broad-based species comparisons across mammalian orders suggest a number of factors that might influence the evolution of large brains. However, the relationship between these factors and total and regional brain size remains unclear. This study investigated the relationship between relative brain size and regional brain volumes and sociality in 13 felid species in hopes of revealing relationships that are not detected in more inclusive comparative studies. In addition, a more detailed analysis was conducted of four focal species: lions (*Panthera leo*), leopards (*Panthera pardus*), cougars (*Puma concolor*), and cheetahs (*Acinonyx jubatus*). These species differ markedly in sociality and behavioral flexibility, factors hypothesized to contribute to increased relative brain size and/or frontal cortex size. Lions are the only truly social species, living in prides. Although cheetahs are largely solitary, males often form small groups. Both leopards and cougars are solitary. Of the four species, leopards exhibit the most behavioral flexibility, readily adapting to changing circumstances. Regional brain volumes were analyzed using computed tomography. Skulls (*n* = 75) were scanned to create three-dimensional virtual endocasts, and regional brain volumes were measured using either sulcal or bony landmarks obtained from the endocasts or skulls. Phylogenetic least squares regression analyses found that sociality does not correspond with larger relative brain size in these species. However, the sociality/solitary variable significantly predicted anterior cerebrum (AC) volume, a region that includes frontal cortex. This latter finding is despite the fact that the two social species in our sample, lions and cheetahs, possess the largest and smallest relative AC volumes, respectively. Additionally, an ANOVA comparing regional brain volumes in four focal species revealed that lions and leopards, while not significantly different from one another, have relatively larger AC volumes than are found in cheetahs or cougars. Further, female lions possess a significantly larger AC volume than conspecific males; female lion values were also larger than those of the other three species (regardless of sex). These results may reflect greater complexity in a female lion’s social world, but additional studies are necessary. These data suggest that within family comparisons may reveal variations not easily detected by broad comparative analyses.

## Introduction

One hypothesis proposed to explain the evolution of large brains is the social brain hypothesis, which posits that social information processing demands enhanced neural computational capabilities leading to larger brains (Dunbar, 1992). In primates, larger brains over and above that expected for a given body size, larger relative neocortical volume and, in particular, larger relative volume of the frontal lobes all positively correlate with social group size (Dunbar and Bever, 1998; Dunbar, 2011). However, whether these findings are unique to primates or apply more generally to other mammalian orders remains unclear. One prediction of the social brain hypothesis is that non-primate species living in large, complex societies also process social information and should exhibit comparable enlargement of brain areas as those found in primate species. The order Carnivora offers a unique opportunity to evaluate the relationship between brain size and sociality since this group consists of more than 280 species in which social complexity ranges from strictly solitary species to highly social species living in large complex groups.

A broad analysis examining brain variation relative to ecological, life history and behavioral factors across Carnivora suggested that a number of factors, most notably, diet and breeding group size, might influence brain size among carnivores (Gittleman, 1986). Support for the social brain hypothesis was provided by three subsequent studies which found positive correlations between sociality in carnivores and relative brain size (Perez-Barberia et al., 2007), relative cerebrum volume (Swanson et al., 2012), and relative neocortex size (Dunbar and Bever, 1998). However, others have emphasized that both social and non-social variables likely influence brain size in carnivores (Holekamp, 2007), including general cognitive abilities as demonstrated by puzzle box problem solving (Benson-Amram et al., 2016). Moreover, Finarelli and Flynn (2009) noted that the positive relationship between sociality and brain size in carnivores is driven mostly by a single family, Canidae, which consists primarily of social species. They found no relationship between brain size and sociality in either Felidae or Hyaenidae (Finarelli and Flynn, 2009). In contrast, a later study that focused on the four extant hyaenid species (Sakai et al., 2011a,b) found that social group size was positively correlated with relative frontal cortex volume. While these last two studies differ somewhat with respect to the variables measured, this discrepancy suggests that analyses at lower taxonomic levels may reveal patterns not easily detectable in broad species comparisons.

Several studies have demonstrated that the relative proportions of brain structures vary uniquely between taxa and suggest that this is a result of grade specific shifts in brain evolution (Barton and Harvey, 2000; Clark et al., 2001; Bush and Allman, 2004; Iwaniuk and Arnold, 2004; Reep et al., 2007; Smaers et al., 2012). It has further been argued that analyses at lower taxonomic levels (e.g., within species and families) may reveal details of brain size variation not observed in comparisons across mammalian orders (Willemet, 2012). Closely related species are expected to possess a characteristic brain cerebrotype, a generalized pattern of weighted brain structures. Deviations from that characteristic cerebrotype can aid in identifying how various selection pressures impact brain evolution. Here, we examine variation in total and regional brain size within the family Felidae in hopes of revealing patterns that are not detected in more inclusive comparative studies.

We investigate the relationship between relative regional brain volumes and sociality in 13 felid species, while controlling for the non-independent effect of phylogeny. In addition, a more detailed species level analysis was conducted of four focal species, including two that differ in sociality by sex (i.e., lions, *Panthera leo*, and cheetahs, *Acinonyx jubatus*) and two that are solitary (i.e., leopards, *Panthera pardus*, and cougars, *Puma concolor*). Lions are the only truly social felids, living in groups (prides) of as many as 21 females and a dominant male (Mosser and Packer, 2009). Females typically stay in the pride to which they were born, while males leave their birth pride and attempt to take over another, often working with other males to overthrow the resident male. The only other social felids are male cheetahs, which often form small groups (Caro and Collins, 1987); female cheetahs, leopards and cougars are typically solitary. This comparison expands on a previous study comparing brain volumes in lions and cougars (Arsznov and Sakai, 2012). Here, three predictions regarding inter- and intraspecific differences and regional brain volumes were evaluated: (1) if sociality presents a greater neural computational demand, then relative brain size is expected to be larger in lions and cheetahs than in other felid species; (2) similarly, since the frontal cortex has been implicated in social information processing, proportionately greater frontal cortex volume should be found in social compared to solitary felids; (3) finally, given that males and females experience very divergent social life histories in lions and cheetahs, but not in leopards and cougars, relative frontal cortex volume should be sexually dimorphic in the former, but not the latter. Here, virtual endocasts were created from computed tomographic (CT) scans of the skulls. Quantitative assessments of total and regional brain volumes were obtained in order to examine both inter- and intra-specific brain variations in selected felid species.

## Materials and Methods

### Sample

A total of 75 adult skulls, representing 13 felid species, were examined. Each species was represented by at least one male (m) and one female (f). Additional skulls were included in a focal species level inquiry: lions (8 males, 6 females), leopards (10 males, 6 females), cougars (6 males, 7 females), and cheetahs (9 males, 5 females). The skulls were obtained from the collections of Michigan State University Museum, University of Michigan Museum of Zoology, American Museum of Natural History and Field Museum of Natural History (see Appendix 1). Data on 11 felid species new to this study are combined with previously collected lion and cougar CT scans (Arsznov and Sakai, 2012). New regional brain volume and skull length data for each skull were collected for this study. Our sampling attempted to avoid, inasmuch as possible, captive status and conspecifics collected from different geographical regions, as captivity and geography are known to contribute to intraspecific variation in brain size in both lions and tigers (Yamaguchi et al., 2009). However, an additional requirement for undamaged skulls with intact tentoriums necessarily limited the selection pool. In our sample, lions, cheetahs and leopards originated primarily from Africa. Although a number of leopard subspecies have been named in Africa, only one (*Panthera pardus pardus*) is supported by genomic analysis (Uphyrkina et al., 2001). Three male leopards were from Sri Lanka and represent an additional leopard subspecies, *Panthera pardus fusca*. Our cougar sample includes individuals from both North and South America. Our analysis did not reveal consistent species level variation related to either captive status or geographical origin.

### Computed Tomography

All skulls were scanned in a General Electric Discovery ST 16 or 64 slice scanner along the rostral-caudal axis in the Department of Radiology, Michigan State University. The following scanning parameters were used: 0.625 mm slice thickness, DFOV 18, 0.531:1 pitch, and 0.5 s scan rotation. Data were saved as Digital Imaging and Communications in Medicine Centricity (DICOM) images and imported to MIMICS 15.0 software (Materialise, Inc., Plymouth, MI, USA), which was used to create the 3D endocasts. These procedures were previously described in detail (Sakai et al., 2011a,b). To summarize, the endocranial air space deep to bone was selected in each coronal section from the cribriform plate rostrally to the opening of the foramen magnum caudally. Sections were subsequently compiled to create a three dimensional virtual endocast. Total endocranial volume and regional volumes were obtained using the MIMICS 3D volume measurement operation.

**FIGURE 1:**
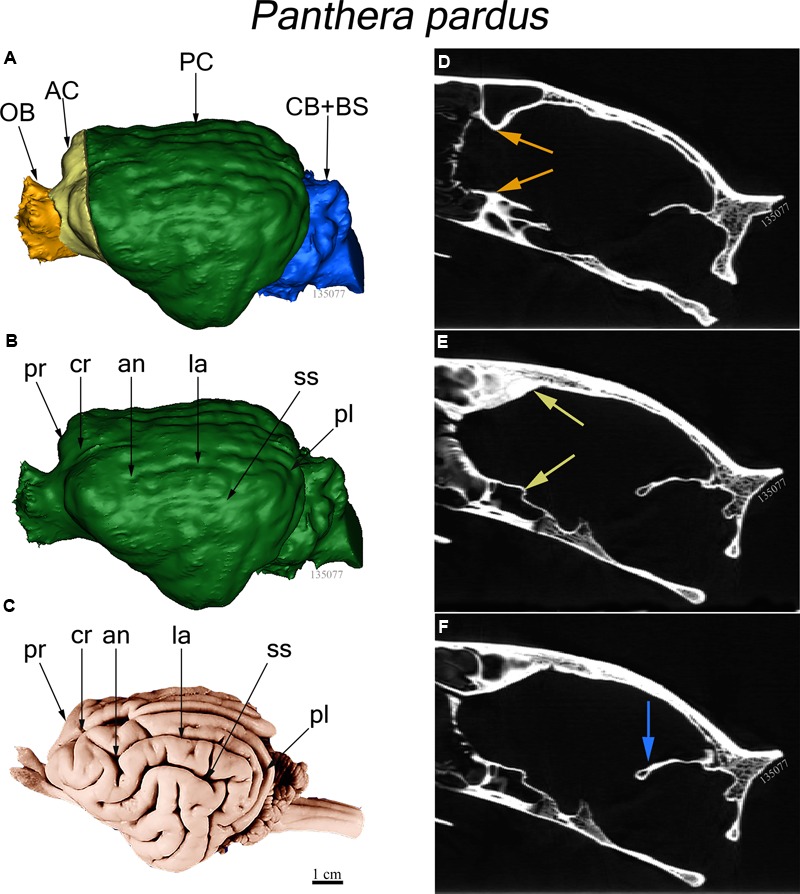
**(A–C)** Three dimensional reconstructions of virtual endocasts from computed tomography (CT) scanned skull and whole brain view of the leopard (*Panthera pardus*). **(A)** Dorsolateral view of a virtual endocast (FMNH specimen 135077) showing the regional brain volumes: Olfactory bulb (OB) in gold, anterior cerebrum (AC) in yellow, posterior cerebrum (PC) in green, and cerebellum and brain stem (CB+BS) in blue. **(B)** Virtual endocast of the same specimen showing the prominent sulci. **(C)** Dorsolateral view of whole brain from the Comparative Mammalian Brain Collection. Abbreviations: an, ansate sulcus; cr, cruciate sulcus; pl, postlateral sulcus; la, lateral sulcus; pr, proreal sulcus; ss, suprasylvian sulcus. Scale bar = 1 cm. **(D–F)** Sagittal CT scans showing bony landmarks used to demarcate brain regions shown in **(A)**. **(D)** Arrows (gold) denote the narrow constriction that demarcates the caudal boundary of OB and the rostral edge of AC. **(E)** Arrows (yellow) indicate the cruciate sulcus at midline (dorsal) and optic canal (ventral), the boundary between caudal AC and rostral PC. **(F)** Arrow (blue) shows the cerebellar tentorium, the boundary separating the caudal PC from rostral CB+BS.

**FIGURE 2:**
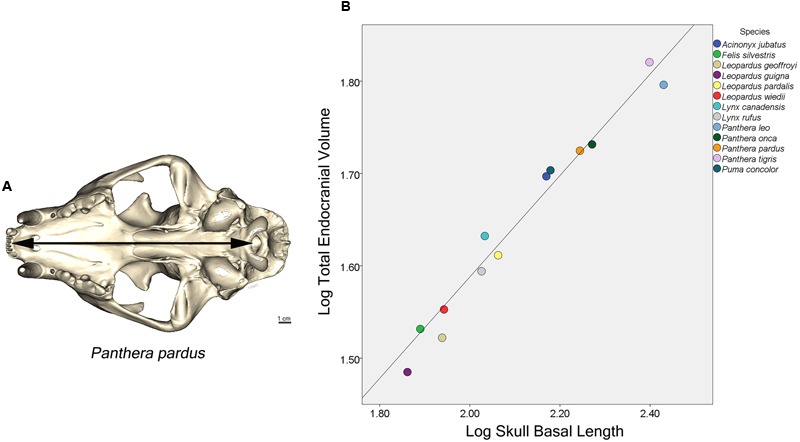
**(A)** Ventral view of a leopard skull showing the linear measure of skull basal length (SBL), the distance from the median incisive alveolus to the mid-ventral border of the foramen magnum (arrow). **(B)** Plot of log cube root of endocranial volume regressed against log skull basal length of 13 felid species. (Pearson’s *r* = 0.96, *p* < 0.001).

The endocast was subdivided into four brain regions based on both bony landmarks and the gyral and sulcal pattern. These subdivisions are: olfactory bulb (OB), anterior cerebrum (AC), posterior cerebrum (PC), and cerebellum/brain stem (CB+BS) (**Figure 1A**). OB volume was defined as the region extending from the cavity posterior to the nasal turbinates to the point of the greatest constriction of this cavity (**Figure 1D**). The region AC extends from OB posteriorly to the cruciate sulcus at midline and ventrally to the optic canal (**Figure 1E**). The cruciate sulcus is used here as a boundary since it is found reliably and is known to coincide with the premotor and motor areas in many carnivores studied (Hassler and Muhs-Clement, 1964; Stanton et al., 1986; Sakai, 1990; Arsznov et al., 2010; Arsznov and Sakai, 2012). Although frontal cortex is defined in primates as the cortex anterior to the central sulcus, the boundary between motor and somatosensory cortex, a similar sulcal boundary is highly variable in both size and shape and is not present in all carnivores (Radinsky, 1969). The AC volume here is thus comprised of the frontal cortex and subcortical structures, including a small portion of the rostral-most head of the caudate nucleus, ventral pallidum, olfactory tubercle and prepiriform cortex. PC volume includes the region caudal to the cruciate sulcus but rostral to the cerebellar tentorium (**Figure 1F**). This region includes cortex caudal to the cruciate sulcus, underlying diencephalic and rostral mesencephalic structures. Lastly, the CB+BS volume lies between the foramen magnum and the cerebellar tentorium. Thus, the CB+BS volume measurement includes cerebellum, caudal mesencephalon, pons and medulla. Body weight data were not available for each individual specimen. Instead, skull basal length, the distance from the anterior border of the median incisive alveolus to the mid-ventral border of the foramen magnum, served as a proxy for body size (**Figure 2A**). Skull basal length strongly correlates with body weight (Radinsky, 1984) and is a reasonable substitute for body size when individual body weight is not available (Janis, 1990; Van Valkenburgh, 1990).

### Statistical Analysis

**FIGURE 3:**
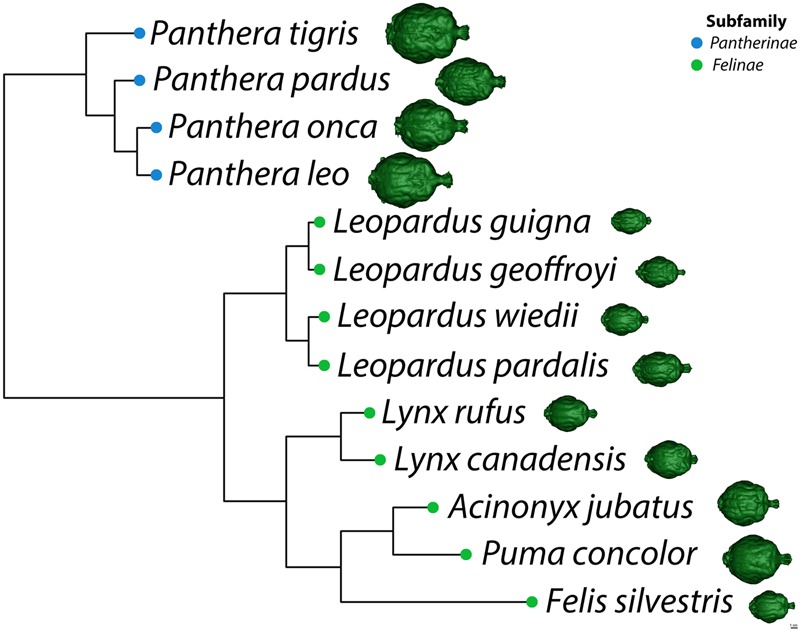
**Phylogeny of selected felid species adapted from Johnson et al. (2006).** A dorsal view of each species’ virtual endocast is shown to scale on the right.

Statistical analysis was based on 75 endocasts representing 13 felid species. The relationship between total endocranial volume and skull basal length was evaluated using Pearson’s *r* correlation coefficient. A phylogenetic least squares regression analysis (PGLS) was performed (Grafen, 1989) on total endocranial volume (species averages) as a function of skull basal length (species averages) and sociality (social vs. solitary). This analysis assumed a Brownian motion model of evolution, using the *nlme* (Pinheiro et al., 2014) and *ape* (Paradis et al., 2004) packages in R version 3.1.0 (R Core Team, 2014). Briefly, a felid phylogeny was constructed for the 13 species using estimated divergence times presented in Johnson et al. (2006) (**Figure 3**). Divergence time for *Felis lybica* was used for *F. silvestris* in our analysis because subspecies information was not available and these skulls originated from Africa. PGLS regressions were also performed on three regional endocranial volumes (AC, PC, and CB+BS), in each case looking at regional volume (species averages) as a function of the rest of endocranial volume (i.e., total endocranial volume minus the volume of that region) and sociality. Additionally, the subfamilies Pantherinae and Felinae (**Figure 3**) were compared with respect to regional endocranial volumes [as a proportion of rest of endocranial volume using phylogenetic analyses of variance (ANOVA) in the geiger package (Harmon et al., 2008)].

For the species-level comparisons of lions, leopards, cougars, and cheetahs, differences in proportional regional brain volumes were examined among species (ANOVA) and between sexes (*t*-tests) using the statistical software package IBM SPSS 20.0 (IBM corporation, Armonk, NY, USA).

## Results

### Interspecies Differences

#### Whole Brain Endocasts

**FIGURE 4:**
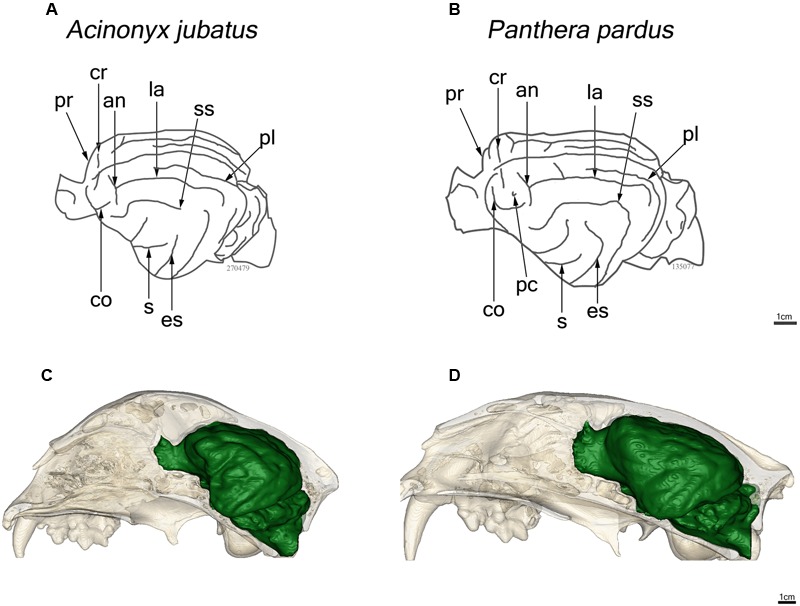
**Line drawings of a lateral view of the endocasts of the cheetah, *Acinonyx jubatus***(A)** and leopard, *Panthera pardus***(B)**.** Abbreviations as in **Figure 1**. *In situ* reconstructions of the skull and endocast for each species are shown in **(C)** and **(D)**.

Major features of external brain morphology are visible in the virtual endocasts, as evidenced by comparing a leopard whole brain photograph (**Figure 1C**) to the virtual endocast from the same species (**Figure 1B**). Prominent sulci including the cruciate sulcus, lateral sulcus, ansate sulcus, suprasylvian sulcus, anterior and posterior limbs of the ectosylvian sulcus and sylvian sulcus are all clearly visible in the endocast (see Figure 1 in Arsznov and Sakai, 2012, for lion and cougar brain-to-endocast comparisons). Gross examination of species differences in brain structure (based on endocasts) indicate that the orientation of, and patterns formed by the sylvian sulcus, suprasylvian sulcus, lateral sulcus, and cruciate sulcus are similar across felids. However, interspecific differences were observed in the presence of small sulci along the midline and in the pattern formed by the postlateral sulcus. Further, while the ectosylvian sulcus divides into an anterior and posterior limb in the same general location in most species studied, it is variably present as a single arched sulcus dorsal to the sylvian sulcus and ventral to the lateral sulcus in lions and cougars. In addition, most felids possess a small post-cruciate sulcus located posterior to the cruciate sulcus and anterior to the limbs of the ansate sulcus, however, this post-cruciate sulcus was not readily identifiable in cheetahs. Line drawings of a dorsolateral view of cheetah and leopard brains are shown in **Figures 4A,B**, respectively. The cruciate sulcus is located quite rostrally and is barely visible in a dorsal view of the cheetah endocast and the most rostral part of the cheetah brain appears flexed in comparison to the more horizontal slope of the dorsal surface of the brain in other felids (see **Figures 4C,D**). When viewed dorsally, the cheetah brain is globose in shape and notably more rounded in appearance than observed in other felids, such as leopards (*Panthera pardus*), ocelots (*Leopardus pardalis*), wildcats (*F. silvestris*), bobcats (*Lynx rufus*), Geoffrey’s cats (*Leopardus geoffroyi*), and kodkods (*Leopardus guigna*) (**Figure 3**).

#### Endocranial Volume and Regional Brain Differences

Skull basal length (the proxy for body size) and total endocranial volume are highly correlated in the 13 felid species examined here (Pearson’s *r* = 0.96, *p* < 0.001) (**Figure 2B**). A PGLS analysis revealed that skull basal length significantly predicts total endocranial volume (β = 0.55, *p* < 0.001), but found no significant effect of sociality on relative endocranial volume (β = 0.28, *p* = 0.22), and no significant interaction (β = -0.13, *p* = 0.20).

**FIGURE 5:**
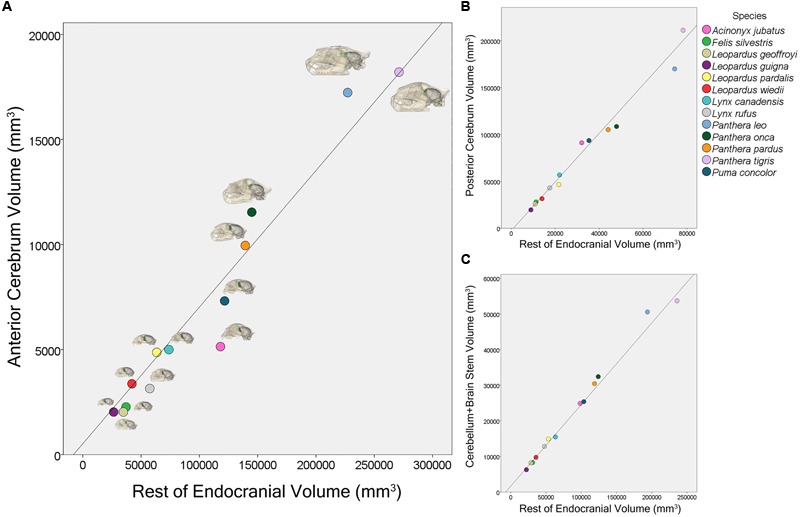
**Scatter plot showing the relationship between anterior cerebrum volume, AC, and rest of endocranial volume **(A)**, posterior cerebrum volume, PC, and rest of endocranial volume **(B)**, and cerebellum and brain stem volume, CB+BS, and rest of endocranial volume **(C)** for 13 felid species**.

Phylogenetic least squares analyses of regional volumes showed that both the rest of endocranial volume (β = 0.065, *p* < 0.001) and the sociality/solitary variable (β = -5891.78, *p* = 0.003) significantly predict AC volume (**Figure 5**). Additionally, there is a significant interaction for AC volume between rest of endocranial volume and sociality/solitary (β = 0.032, *p* = 0.004) wherein rest of endocranial volume is a significant predictor of AC volume in solitary species. Rest of endocranial volume also significantly predicts PC volume (β = 2.60, *p* < 0.001) and CB+BS volume (β = 0.23, *p* < 0.001) (**Figure 5**). However, sociality was not a significant predictor in either analysis: PC (β = 23264.90, *p* = 0.07), CB+BS volumes (β = -1521.80, *p* = 0.60). Averaged endocranial and regional brain volumes for each species are shown in **Table 1**.

**Table 1:** Average endocranial and regional brain volumes by species.

Species	Average Volume (mm^3^)			
	Total endocranium	Anterior cerebrum	Posterior cerebrum	Cerebellum + Brain stem
*Acinonyx jubatus*	123273.85	5142.89	91175.42	24883.56
*Lynx rufus*	60584.94	3145.84	43061.71	12817.80
*Felis silvestris*	39346.93	2268.24	28035.93	8268.72
*Leopardus geoffroyi*	36842.94	2026.78	25939.62	8185.28
*Leopardus guigna*	28505.60	2028.27	19540.50	6258.30
*Leopardus pardalis*	68282.95	4866.14	46593.10	14905.56
*Leopardus wiedii*	45526.52	3371.04	31506.63	9745.92
*Lynx canadensis*	78873.34	4999.91	56902.51	15459.24
*Panthera leo*	244416.26	17231.45	170056.34	50545.21
*Panthera onca*	156479.09	11540.05	108563.60	32399.69
*Panthera pardus*	149319.62	9954.66	105203.34	30425.05
*Panthera tigris*	289463.72	18206.34	211268.89	53660.62
*Puma concolor*	128957.55	7316.05	93589.41	25374.98

Phylogenetic ANOVAs found no significant difference between subfamilies Felinae and Pantherinae in relative proportions of AC [*F*(1,11) = 2.08, *p* = 0.69], PC [*F*(1,11) = 0.05, *p* = 0.94)], or CB+BS [*F*(1,11) = 2.43, *p* = 0.65].

### Regional Brain Volume Differences in Four Felid Species

**FIGURE 6:**
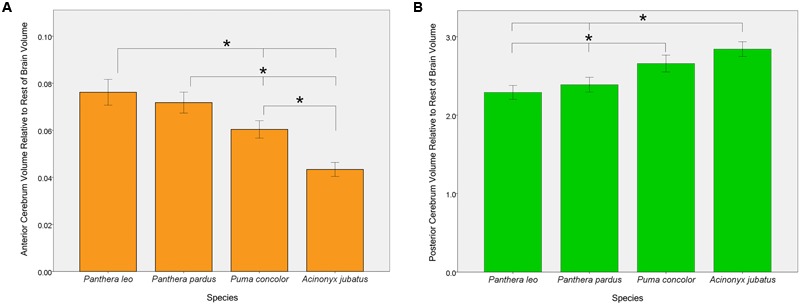
**Pairwise comparisons of anterior cerebrum volume, AC **(A)** and posterior cerebrum volume, PC **(B)** relative to the rest of endocranial volume in four felid species.** Bars indicate 95% CI. Lions and leopards have significantly larger relative ACs than either cougars (*p* < 0.001, *p* = 0.001, respectively) or cheetahs (*p* < 0.001, *p* < 0.001, respectively), and cougars have a significantly larger relative AC than cheetahs (*p* < 0.001). Although relative AC volume is greater in lions than in leopards, this difference is not statistically significant (*p* = 0.71). Relative PC volume is significantly greater in cheetahs and cougars than in lions (*p* < 0.001, *p* < 0.001, respectively) or leopards (*p* < 0.001, *p* < 0.001, respectively), but does not differ significantly between lions and leopards (*p* = 0.74) or between cougars and cheetahs (*p* = 0.05).

One-way ANOVAs comparing the four focal species (lions, leopards, cougars, and cheetahs) yielded significant effects of both AC and PC to the rest of endocranial volume [*F*(3,53) = 53.60, *p* < 0.001 and *F*(3,53) = 30.72, *p* < 0.001, respectively] (**Figure 6**). Bonferroni *post hoc* comparisons revealed that both lions and leopards have a significantly larger relative AC than cougars (*p* < 0.001, *p* = 0.001, respectively) or cheetahs (*p* < 0.001, *p* < 0.001, respectively). Additionally, while lions have a relatively larger AC than leopards, this difference is not statistically significant (*p* = 0.71). Cheetahs have the smallest AC to rest of endocranial volume of the four species (*p* < 0.001; **Figure 6**). *Post hoc* comparisons of relative PC to rest of endocranial volume revealed that both cougars and cheetahs have a significantly larger relative PC than lions (*p* < 0.001, *p* < 0.001, respectively) or leopards (*p* < 0.001, *p* < 0.001, respectively). However, there were no significant differences in relative PC between cougars and cheetahs (*p* = 0.05) or between lions and leopards (*p* = 0.74). No significant main effect of the relative volume of CB+BS was present between the species [*F*(3,53) = 1.34, *p* = 0.27].

#### Sex Differences in Regional Brain Volume in Four Felid Species

**FIGURE 7:**
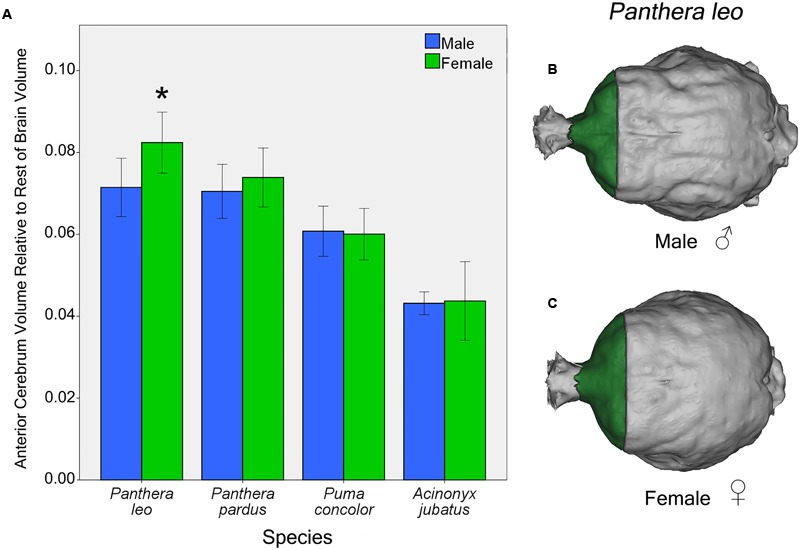
**Sex differences in AC volume relative to rest of endocranial volume in four felid species **(A)**.** Females lions possess a significantly larger relative AC than males [*t*(12) = 2.54, *p* = 0.03]. Relative AC volume did not differ by sex in the leopard, cougar or cheetah. Dorsal view of male **(B)** and female **(C)** lion endocasts. Bars indicate 95% CI.

Independent *t*-tests for sex differences in proportional regional brain volumes of the four focal species (lions, leopards, cougars, and cheetahs) revealed significant sex differences in lions and cougars, but not in cheetahs or leopards. In lions, females possess a significantly larger AC to rest of endocranial volume than males [*t*(12) = 2.54, *p* = 0.03] (**Figure 7A**). This difference is evident from gross examination of the dorsal brain surface of adult male and female lions (**Figures 7B,C**). No significant sex differences in relative PC or CB+BS were found in lions [*t*(12) = 0.57, *p* = 0.58 and *t*(12) = 0.68, *p* = 0.51, respectively]. For cougars, no significant sex difference was identified in relative AC [*t*(11) = 0.20, *p* = 0.84], but females had a significantly larger relative PC than males [*t*(11) = 3.32, *p* = 0.007], while males had a significantly larger relative CB+BS than females [*t*(11) = 2.67, *p* = 0.02].

## Discussion

The relationship between regional brain volumes and sociality was examined in wild-caught representatives of 13 felid species. We found that sociality does not correspond with larger relative brain size in these species. However, a PGLS regression analysis revealed that the interaction of the sociality/solitary variable and rest of endocranial volume has a significant effect on AC volume. Relative AC volume is significantly predicted by rest of endocranial volume in solitary species. The two social felid species, lions and cheetahs, possess the largest and smallest relative AC volumes, respectively. Inter- and intra-specific comparisons of regional brain volumes in four focal species (lions, leopards, cougars, and cheetahs) revealed that lions and leopards have the largest relative AC volumes, and lions exhibit significant sexual dimorphism in relative AC, with larger volumes in females than in males. Based on these results, the functional role in felid behavior of frontal cortex, a major component of AC volume, remains largely unknown and requires further investigation. Nonetheless, these data suggest that within family and within species comparisons reveal patterns not easily detected by broader comparative analyses.

### Toward a Felid Cerebrotype

Our observations of a general felid cerebrotype, reflected in characteristic regional brain morphology, is consistent with the remarkable similarities in behavior, diet and ecology noted across the family Felidae (Eaton, 1979; Bekoff et al., 1984; Mellen, 1993). Most member species are found in woodland or woodland fringe terrain, most are nocturnal, most are meat eaters and typically stalk and ambush prey (Eaton, 1979; Bekoff et al., 1984; Mellen, 1993). An inspection of the endocasts and 3D printed models created for each of the 13 species revealed remarkable similarity in gross external brain morphology. The general pattern formed by sulci and gyri exhibited little variation despite a large range in brain size and species relatedness. The configuration and location of major sulci including the lateral sulcus, suprasylvian sulcus, and sylvian sulcus were very similar across species (**Figure 3**). For example, the anterior and posterior limbs of the ectosylvian sulcus typically remain separate in felids and the pattern formed by, and orientation of, the cruciate sulcus are also relatively constant. The cruciate sulcus is an important sulcus delimiting caudal area 4, the primary motor cortex, from rostral area 4 and the premotor area 6 based on cytoarchitectonic analysis in the cat (Hassler and Muhs-Clement, 1964), dog (Tanaka et al., 1981; Stanton et al., 1986), ferret (McLaughlin et al., 1998), and raccoon (Sakai, 1990). Although this is a limited sample, it is reasonable to infer that the cruciate sulcus similarly demarcates the premotor and rostral motor areas from the caudal motor area in wild felids. Another relatively constant feature of the felid pericruciate region found in most of the studied species is the presence of a post-cruciate sulcus, a small sulcus located posterior to the cruciate sulcus and rostral to the medial and lateral limbs of the ansate sulcus. This sulcus is often quite variable in carnivores, but when present, demarcates the boundary between the somatosensory cortex caudally and the motor cortex rostrally based on electrophysiological mapping studies in the cat (Nieoullon and Rispal-Padel, 1976), dog (Gorska, 1974), and raccoon (Welker and Seidenstein, 1959; Hardin et al., 1968). The remarkable similarities in brain morphology and sulcal patterns among extant felids were previously noted by Radinsky (1969, 1975) and our present observations largely support his descriptions.

Some deviations from the general felid plan were also noted. For example, although the two limbs of the ectosylvian sulcus are typically separate, it is a single complete arch uniting the anterior and posterior ectosylvian sulci inconsistently either between cerebral hemispheres or between individuals in lions and less often in cougars. The middle ectosylvian gyrus contains the primary auditory cortex in the cat (Reale and Imig, 1980) and dog (Tunturi, 1962; Lyras and Van Der Geer, 2003), but it is unclear how the presence of a single complete ectosylvian arch in these species might be functionally related. Second, overall brain shape ranged from a rounded, globose morphology, to a more elongated form across the felid species. In the globose form, the temporal lobes appear more distinct and broader than those found in the elongated form. Radinsky (1975) noted the globose brain shape of cheetahs and *Lynx* sp. (including *Lynx canadensis*) and suggested that this form may be more characteristic of smaller felids. Our data confirm that the globose form is found in cheetahs and *Lynx canadensis*, but also in lions, tigers, cougars, and jaguars. A more elongated brain shape is evident in leopards, ocelots, wildcats, bobcats, Geoffrey’s cats and kodkods. Whether this difference in brain shape is related to felid brain evolution or a consequence of variation in skull morphology is beyond the scope of the present study. However, a recent analysis using geometric morphometrics to examine brain shape evolution in New World monkeys (Aristide et al., 2016) promises to be a useful method in future studies examining convergence of brain phenotypes.

### Exceptions to the Rule: Cheetahs and Lions

Despite the relative conservation of behavior, diet, and ecology, along with the remarkable similarities in sulcal and gyral patterns and overall brain shape among felids, two species stand out from the others: cheetahs and lions. These are the only social species in the family, and while relative AC volume is predicted by rest of endocranial volume for solitary species, that was not the case for these social cats. Relative regional brain volumes differ significantly between lions and cheetahs, and also between each of these species and other felids.

The brain morphology of cheetahs is distinctive in at least three respects. First, the post-cruciate sulcus that typically separates motor and somatosensory cortex in felids is absent in cheetahs. Radinsky (1975) first noted this difference and speculated that the absence of this sulcus might be related to limited motor control of limb musculature. Cheetahs are limited in forepaw use (Van Valkenburgh, 1996). The ability to climb trees and manipulate prey is diminished compared to other felids. In tests of forepaw dexterity, cheetahs rank the lowest among felids studied (Iwaniuk et al., 2000). Second, overall brain shape is more globular in cheetahs than in other felids; this appears to reflect the broad expanse of the temporal lobes. Finally, the brain of cheetahs exhibits a distinctive rostral dorsiflexion not found in other cats (**Figures 3** and **4C**). This dorsiflexion and the associated domed skull likely reflect the cheetah’s unusually large frontal sinuses (O’Regan, 2002; Sicuro and Oliveira, 2011; Curtis and Van Valkenburgh, 2014), which are hypothesized to act as a vascular cooling mechanism facilitating the ability to engage in high speed chases (Salles, 1992; Sicuro and Oliveira, 2011). The unique skull morphology of cheetahs is also thought to improve biting efficiency, allowing the animal to use less muscle force to achieve killing bite strength (Chamoli and Wroe, 2011; Sicuro and Oliveira, 2011). The reduced muscle and bone mass contribute to a lighter body, which may also facilitate increased speed in chase pursuits.

Social life history is sexually divergent in cheetahs. As in most other felids, female cheetahs are typically solitary except when raising young. However, male cheetahs are considered semi-social. They may be solitary (41%) or form coalitions comprised of two (40%) or three (19%) (usually related) individuals (Caro and Collins, 1987; Durant et al., 2004). These male coalitions can last a lifetime. Male coalitions gain a territorial and reproductive advantage in regions where coalitions are common (Durant et al., 2004). Although male coalitions may hunt larger prey than solitary cheetahs, they cooperate little during a hunt (Caro and Kelly, 2001). We expected that the social behavior exhibited by male cheetahs might be reflected in its having a larger relative AC volume than non-social cats (including female cheetahs), similar to the effect found previously in social carnivores such as spotted hyenas (Arsznov et al., 2010), coatimundis (Arsznov and Sakai, 2013), and lions (Arsznov and Sakai, 2012). However, we found that cheetahs possess the smallest anterior cerebrum volume of all species studied here and exhibit no significant sexual dimorphism.

Cheetahs also possess the smallest relative brain volume of the species we sampled. Small brains weigh less and require less energy, factors that might contribute to the remarkable running speeds that cheetahs achieve. The unique morphology of cheetahs might also reflect, at least in part, their unusual genetic history. Cheetahs experienced two severe population bottlenecks, one as many as 10,000 years ago, and a second, as recently as within the last 100 years (O’Brien et al., 1987), resulting in a 95–98% reduction in genetic diversity compared to other (selected) wild felids (Dobrynin et al., 2015). In our sample, the closest relative to cheetahs, cougars, possess both larger relative brain size and larger relative AC volume.

The largest relative AC volume was found in female lions, which are, by far, the most gregarious of felids. The social group, the pride, consists of as many as 21 adult females, offspring and male coalitions (Mosser and Packer, 2009; VanderWaal et al., 2009). Female lions are primarily philopatric and remain social throughout their lives. The pride is a stable social unit lacking a dominance hierarchy. Pride females communally rear their young and may nurse offspring of other pride members. They work together to protect cubs from invading males (Packer et al., 2001) and will also coordinate their movements in hunting large prey and defending their territory (Packer and Pusey, 1997). Male lions, in contrast, disperse upon reaching sexual maturity and live alone or as part of a small coalition of usually brothers or cousins (Packer and Pusey, 1997; VanderWaal et al., 2009). A nomadic male lion may challenge a dominant male to take over a pride, then aggress, often lethally, against young cubs. Typically, a male lion is dominant in the pride for 2–3 years before being displaced. Solitary male lions are less successful hunters than group hunting female lions. Although the lion pride is a fission–fusion society with individuals dispersing and returning to the group (VanderWaal et al., 2009), it is socially cohesive. Social network analysis of both captive origin and wild prides indicate that female lions are central to the social network with adult male lions most likely to receive and least likely to initiate social interactions (Dunston et al., 2016a,b). Male lions are not only less social than female lions, but are also larger and more aggressive than female lions. The cognitive demands associated with social information processing are expected to be greater in female than male lions. Appropriate behavior in response to dominance and aggression demands impulse control and behavioral inhibition. These behaviors are associated with frontal cortex functions in other species, particularly primates (Hauser, 1999; for review, see Passingham and Wise, 2012). Although the role of frontal cortex in felids is not yet known, the finding of a larger relative AC volume in female lions than in other cats (including male lions) might similarly reflect the unusual social demands they encounter.

An association between relative AC volumes and sociality has been found in other carnivore families. The highly social spotted hyena has a larger relative AC volume than other, less social hyenids (Sakai et al., 2011a,b). Similarly, a larger relative AC volume was found in the social coatimundis than in less social procyonids (Arsznov and Sakai, 2013). Moreover, sex differences in relative AC volume were found in both spotted hyenas (Arsznov et al., 2010) and coatimundis (Arsznov and Sakai, 2013), consistent with enhanced social processing demands.

Despite much inquiry, it remains unclear whether enhanced cognitive skills or the cognitive demands of social information processing selectively drive frontal cortex size and/or relative or absolute brain size (Gittleman, 1986; Dunbar, 2011; Smaers et al., 2012; MacLean et al., 2014). A recent study examined performance in 39 carnivore species across nine families and reported that problem solving capabilities corresponded with larger relative brain size but failed to find an association with sociality (Benson-Amram et al., 2016). However, large-scale comparisons can overlook variation occurring at a family level. A similar problem-solving task was applied to study social and solitary felids and found that lions exceeded the performance of leopards and tigers on measures of innovation and exploration in solving a puzzle box problem (Borrego and Gaines, 2016). These authors suggest that the superior cognitive performance exhibited by lions is related to selection pressures associated with sociality. In this regard, it is of interest that here relative AC volume in leopards, a solitary species, did not differ significantly from that found in lions, and was larger than that found in cheetahs, suggesting that factors other than sociality drive AC volume. Of all the wild felids, leopards are especially noted for their behavioral flexibility and adaptability (Hayward and Kerley, 2008; Pitman et al., 2013), behaviors associated with enhanced cognitive processing (Sol, 2009). These results suggest that taxon level analysis as well as within species comparisons lead to results not easily detected by broader comparative analyses. Additional studies examining social, ecological and life history variables associated with regional brain volume differences within carnivoran families are needed. Other carnivoran families such as Mustelidae, Canidae, and Herpestidae exhibit greater variation in social behavior than that found in Felidae. Parallel regional brain analyses within these families are expected to further elucidate the association between sociality and relative frontal cortex volume.

## Author Contributions

Conceived and designed study: SS and BL. Collected, analyzed, and interpreted data: AH, EY, BA, BL, and SS. Wrote, revised, and edited: SS, BL, BA, AH, and EY.

## Conflict of Interest Statement

The authors declare that the research was conducted in the absence of any commercial or financial relationships that could be construed as a potential conflict of interest.
