# Supplementary material for: Big Cat Coalitions: A Comparative Analysis of Regional Brain Volumes in Felidae
Source: Front Neuroanat. 2016 Oct 20;10:99. doi: 10.3389/fnana.2016.00099 (PMC5071314; doi:10.3389/fnana.2016.00099)
Supplement: Supplementary file 1 [file Data_Sheet_1.docx]

Appendix 1.

| **Museum** | **Catalog No.** | **Species** | **Sex** | **Museum** | **Catalog No.** | **Species** | **Sex** |
| --- | --- | --- | --- | --- | --- | --- | --- |
| FMNH | 29634 | *Acinonyx jubatus raineyii* | F | MSU | 25115 | *Lynx canadensis* | M |
| FMNH | 29635 | *Acinonyx jubatus raineyii* | M | MSU | 11245 | *Panthera pardus* | F |
| FMNH | 34589 | *Acinonyx jubatus jubatus* | M | MSU | 27989 | *Panthera pardus* | M |
| FMNH | 60535 | *Acinonyx jubatus* | M | MSU | 3949 | *Panthera leo* | M |
| FMNH | 104598 | *Acinonyx jubatus* | F | MSU | 8046 | *Panthera leo* | F |
| FMNH | 104810 | *Acinonyx jubatus* | M | MSU | 11241 | *Panthera leo* | M |
| FMNH | 127834 | *Acinonyx jubatus raineyii* | F | MSU | 11242 | *Panthera leo* | M |
| FMNH | 150779 | *Acinonyx jubatus* | M | MSU | 11675 | *Panthera leo* | M |
| FMNH | 20762 | *Panthera leo* | M | MSU | 14954 | *Panthera leo* | F |
| FMNH | 20756 | *Panthera leo* | F | MSU | 21884 | *Panthera leo* | M |
| FMNH | 20758 | *Panthera leo* | F | MSU | 24411 | *Panthera leo* | M |
| FMNH | 1446 | *Panthera pardus nanopardus* | F | MSU | 29988 | *Panthera leo* | M |
| FMNH | 27006 | *Panthera pardus adusta* | M | MSU | 36073 | *Panthera leo* | F |
| FMNH | 27279 | *Panthera pardus suahelica* | M | MSU | 2876 | *Panthera onca* | M |
| FMNH | 27443 | *Panthera pardus fusca** | M | MSU | 12243 | *Panthera onca* | F |
| FMNH | 32943 | *Panthera pardus adusta* | M | MSU | 11679 | *Panthera tigris* | F |
| FMNH | 34590 | *Panthera pardus shortridgei* | F | MSU | 16626 | *Panthera tigris* | M |
| FMNH | 34591 | *Panthera pardus shortridgei* | M | MSU | 3810 | *Puma concolor* | F |
| FMNH | 60626 | *Panthera pardus saxicolor* | F | MSU | 3811 | *Puma concolor* | F |
| FMNH | 99534 | *Panthera pardus fusca* | M | MSU | 6321 | *Puma concolor* | F |
| FMNH | 99535 | *Panthera pardus fusca* | M | MSU | 6322 | *Puma concolor* | F |
| FMNH | 99536 | *Panthera pardus fusca* | M | MSU | 6325 | *Puma concolor* | F |
| FMNH | 127842 | *Panthera pardus suahelica* | F | MSU | 10658 | *Puma concolor* | F |
| FMNH | 135075 | *Panthera pardus suahelica* | F | MSU | 10659 | *Puma concolor* | M |
| FMNH | 135077 | *Panthera pardus suahelica* | M | MSU | 12240 | *Puma concolor* | M |
| MSU | 8047 | *Acinonyx jubatus* | M | MSU | 12387 | *Puma concolor* | F |
| MSU | 35116 | *Felis rufus** | M | MSU | 13071 | *Puma concolor* | M |
| MSU | 35210 | *Felis rufus** | F | MSU | 14361 | *Puma concolor* | M |
| MSU | 24298 | *Felis silvestris* | F | MSU | 14363 | *Puma concolor* | M |
| MSU | 24300 | *Felis silvestris* | M | MSU | 35996 | *Puma concolor* | M |
| MSU | 17866 | *Leopardus geoffroyi* | M | NMNH | 163090 | *Acinonyx jubatus raineyii* | M |
| MSU | 2116 | *Leopardus guigna* | M | NMNH | 173001 | *Acinonyx jubatus raineyii* | M |
| MSU | 6313 | *Leopardus guigna* | F | NMNH | 251793 | *Acinonyx jubatus* | F |
| MSU | 14592 | *Leopardus pardalis* | F | NMNH | 270479 | *Acinonyx jubatus* | M |
| MSU | 14593 | *Leopardus pardalis* | M | NMNH | 395137 | *Acinonyx jubatus* | F |
| MSU | 14609 | *Leopardus wiedii* | F | UMMZ | 146504 | *Leopardus geoffroyi* | F |
| MSU | 14727 | *Leopardus wiedii* | M | UMMZ | 114803 | *Panthera leo* | F |
| MSU | 7754 | *Lynx canadensis* | F |  |  |  |  |

**Felis rufus* has been re-classified as *Lynx rufus*.

Field Museum (FMNH), Michigan State University Museum (MSUM), University of Michigan Museum of Zoology (UMMZ), National Museum of Natural History (NMNH)
